# Complete Resection of a Giant Hypervascular Pelvic Floor Solitary Fibrous Tumor Using Intraoperative Balloon Occlusion and Staged Open Abdomen Management: A Case Report

**DOI:** 10.70352/scrj.cr.25-0717

**Published:** 2026-03-05

**Authors:** Masahiro Hashimoto, Taishi Hata, Hiroki Akashi, Shinya Kato, Yoshihiro Morimoto, Yujiro Nishizawa, Kenta Furukawa, Miho Yamakawa, Tetsuro Nakazawa, Keiko Matsuoka, Kohki Shimazu, Akira Tomokuni, Masaaki Motoori, Kazumasa Fujitani

**Affiliations:** 1 Department of Gastroenterological Surgery, Osaka General Medical Center, Osaka, Osaka, Japan; 2 Department of Diagnostic and Interventional Radiology, Osaka General Medical Center, Osaka, Osaka, Japan; 3 Department of Pathology, Osaka General Medical Center, Osaka, Osaka, Japan

**Keywords:** solitary fibrous tumor, pelvic floor, internal iliac vessels, balloon occlusion, open abdomen management

## Abstract

**INTRODUCTION:**

Solitary fibrous tumors (SFTs) are rare fibroblastic neoplasms that can occur at various anatomical sites, including the pleura, retroperitoneum, and pelvis. Although surgical resection remains the mainstay of curative treatment, pelvic SFTs often present as giant hypervascular tumors, making intraoperative bleeding control particularly challenging.

**CASE PRESENTATION:**

A 66-year-old man presented with progressive abdominal distension, constipation, and dysuria. CT revealed a large pelvic mass measuring 200 × 176 × 140 mm, with multiple intratumoral vessels and areas of necrosis accompanied by bilateral hydronephrosis. MRI revealed a heterogeneously hyperintense signal on T2-weighted images. Preoperative angiography revealed multiple feeding arteries from the bilateral internal iliac, inferior mesenteric, and median sacral arteries. Preoperative embolization was deemed technically difficult because of the extensive vascular network. Intraoperative balloon occlusion catheters were therefore placed in both internal iliac arteries to control pelvic blood flow. The tumor was resected via open surgery, along with partial cystectomy and ureteral resection, followed by reconstruction. Persistent venous oozing required temporary open abdomen management using Abthera, and definitive closure was achieved the following day after confirmation of hemostasis and application of Surgiflo. The resected specimen measured 210 × 200 × 140 mm and weighed 2865 g. Histologically, the tumor consisted of spindle cells with low mitotic activity. Immunohistochemistry revealed positivity for signal transducer and activator of transcription 6 (STAT6) and negativity for cluster of differentiation 34 (CD34), confirming the diagnosis of SFT. The postoperative course was complicated by pulmonary embolism, which was successfully managed with anticoagulation therapy. The patient remains disease-free 1 month after surgery.

**CONCLUSIONS:**

This case of a giant pelvic floor SFT with CD34 negativity and STAT6 positivity demonstrates that intraoperative balloon occlusion and staged open abdominal management can be effective strategies for controlling intraoperative bleeding in hypervascular pelvic tumors. Individualized planning and staged approaches are crucial for facilitating tumor resection in such highly challenging cases.

## Abbreviations

CD34
: cluster of differentiation 34
OAM
: open abdominal management
SFT
: solitary fibrous tumor
STAT6
: signal transducer and activator of transcription 6
TAE
: transcatheter arterial embolization

## INTRODUCTION

SFTs are hypervascular, clinically rare fibroblastic neoplasms. They were first described by Klemperer and Rabin in 1931 and have since been reported in various anatomical sites, including the pleura, retroperitoneum, and abdominopelvic cavity.^1)^ Complete surgical resection is considered the most effective curative treatment. However, when SFTs arise in the pelvic floor, they often become large, and intraoperative bleeding control becomes a critical challenge.^2,3)^ Herein, we report a case of a giant pelvic floor SFT that was successfully resected using a combined strategy of intraoperative balloon occlusion and staged OAM.

## CASE PRESENTATION

A 66-year-old man was referred to our hospital with a chief complaint of lower abdominal distension. He had noticed abdominal fullness 1 month earlier, followed by constipation and dysuria during the subsequent 2 weeks. His medical history included prostate cancer treated with radiotherapy 7 years earlier, with no recurrence to date, diabetes mellitus, and glaucoma. His family history included pancreatic cancer in his grandfather, gastric cancer in his grandmother, and breast cancer in his sister. On physical examination, a firm, immobile mass was palpable in the lower abdomen below the umbilicus, and swelling of the left lower limb was observed. Laboratory tests showed preserved renal function, and the levels of tumor markers, including CEA and CA19-9, were within normal limits. Lower-extremity duplex ultrasonography revealed no evidence of deep venous thrombosis.

**Fig. 1:**
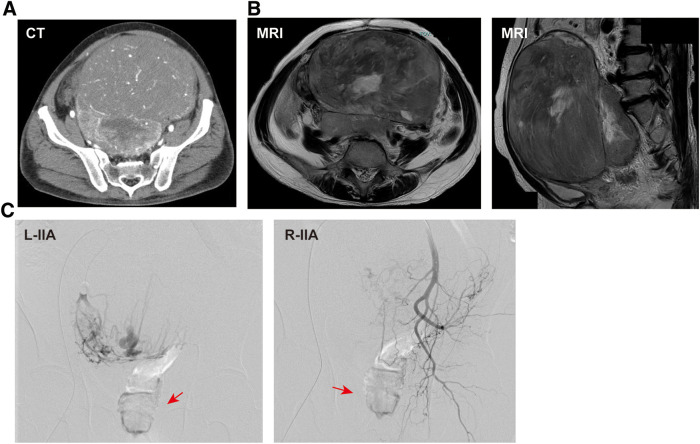
Preoperative imaging findings. (**A**) CT showing a 200 × 176 × 140 mm pelvic mass with intratumoral vessels and necrosis. (**B**) T2-weighted MRI demonstrating heterogeneous hyperintensity. (**C**) Angiography showing tumor hypervascularity with major feeders from the bilateral internal iliac arteries. The tumor is indicated by red arrows. IIA, internal iliac arteries; L, left; R, right

**Fig. 2:**
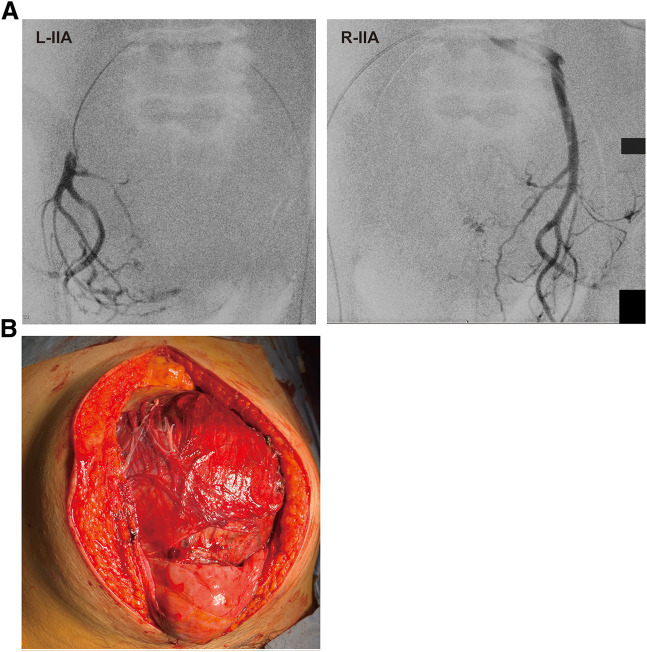
Intraoperative findings. (**A**) Bilateral internal iliac balloon occlusion for temporary control of pelvic blood flow. (**B**) Exposed hypervascular pelvic tumor requiring partial cystectomy and partial right ureteral resection. IIA, internal iliac arteries; L, left; R, right

CT revealed a large soft-tissue mass measuring approximately 200 × 176 × 140 mm, occupying the pelvic cavity and extending into the lower abdomen (**Fig. 1A**). The mass contained multiple intratumoral vessels and areas of low attenuation consistent with necrosis. MRI showed a heterogeneously hyperintense signal on T2-weighted images, reflecting the tumor’s rich vascularity and internal necrotic changes (**Fig. 1B**). Bilateral hydronephrosis caused by ureteral compression was also evident on preoperative imaging (**Supplementary Fig. 1A**). Preoperative angiography revealed tumor hypervascularity with multiple feeding arteries arising from the bilateral internal iliac arteries, right external iliac artery, lumbar artery, and inferior mesenteric artery, indicating a complex vascular supply (**Fig. 1C** and **Supplementary Fig. 1B–1D**). The predominant venous drainage of the tumor was via the bilateral internal iliac venous systems. Because of this extensive vascularization, preoperative embolization was considered technically difficult and was not performed. Instead, balloon occlusion catheters were placed in both internal iliac arteries via a bilateral inguinal approach immediately before surgery to control intraoperative bleeding (**Fig. 2A**). During the dissection of the hypervascular pelvic floor region, the balloons were intermittently inflated for 30 min, followed by 10 min of deflation in repeated cycles to maintain adequate hemostasis while minimizing ischemic injury to the pelvic organs. Ureteral stenting for bilateral hydronephrosis was attempted but failed because of tumor compression.

Considering the tumor size and hypervascularity, open surgery was performed (**Fig. 2B**). Partial cystectomy and resection of the right ureter were performed, followed by reconstruction by a urology team. The operative time was 7 h 23 min. Due to concomitant urinary tract reconstruction, accurate blood loss measurement was not feasible; however, massive transfusion was required, including 48 units of red cell concentrate and 64 units of fresh-frozen plasma. Bleeding from the draining veins connected to the right internal iliac vein was observed, and hemostasis was achieved by coagulation and compression. Persistent minor oozing necessitated placement of packing gauze and temporary open abdomen management using Abthera (Acelity, San Antonio, TX, USA). The patient was transferred to the ICU.

Postoperative laboratory examinations demonstrated stable coagulation parameters and blood cell counts, with no evidence of progressive coagulopathy, allowing definitive abdominal closure on the following day. Second-look laparotomy revealed no active bleeding. After topical application of Surgiflo (Ethicon, Somerville, NJ, USA), the abdomen was closed. The patient was extubated and transferred to the ICU on POD 1, where oral intake was resumed.

**Fig. 3:**
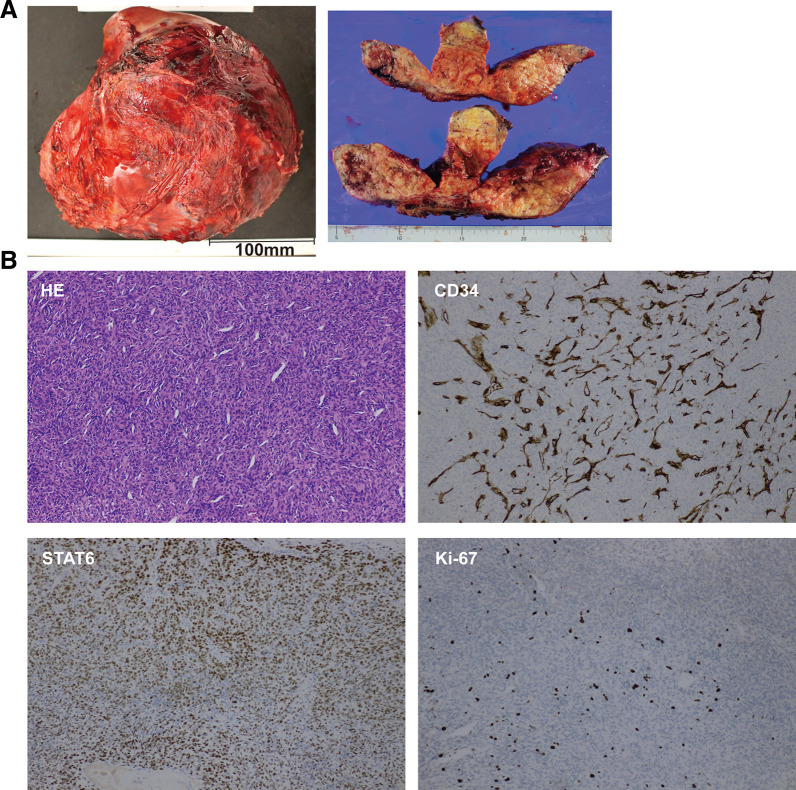
Pathological findings. (**A**) Resected 210 × 200 × 140-mm tumor showing hemorrhage and necrosis. (**B**) Histopathology: H&E staining shows densely proliferating spindle cells with low mitotic activity; immunohistochemistry demonstrates nuclear STAT6 positivity, loss of CD34 expression, and a Ki-67 labeling index of approximately 6%, consistent with SFT. All images were taken at ×100 magnification. CD34, cluster of differentiation 34; H&E, hematoxylin–eosin; SFT, solitary fibrous tumor; STAT6, signal transducer and activator of transcription 6

The resected tumor measured 210 × 200 × 140 mm and weighed 2865 g. The cut surface was gray-white and solid, with extensive hemorrhage and necrosis (**Fig. 3A**). Histologically, short spindle-shaped cells proliferated densely with sparse mitotic figures (0–1 per 10 high-power fields) (**Fig. 3B**). Immunohistochemically, the tumor cells were negative for CD34, positive for STAT6, focally positive for S-100, weakly positive for cytokeratin (AE1/AE3), and negative for CD117, DOG1, SOX10, HMB-45, Desmin, SMA, MDM2, and CDK4. The Ki-67 labeling index was 6% (**Fig. 3B** and **Supplementary Fig. 2**). Based on these findings, the tumor was diagnosed as an SFT despite CD34 negativity.

Postoperatively, a pulmonary embolism was detected on follow-up CT, and anticoagulation therapy was initiated. The clinical course was uneventful, and the patient was discharged on POD 16. ^18^F-fluorodeoxyglucose PET-CT performed 1 month after surgery showed no abnormal uptake, and the patient remains alive without recurrence.

## DISCUSSION

SFTs are rare fibroblastic neoplasms that account for less than 2% of all soft tissue tumors. Although initially described in the pleura, extrapleural SFTs represent approximately one-third of cases, with the retroperitoneum and pelvis being particularly uncommon sites.^1–3)^ Pelvic SFTs often remain asymptomatic until they reach a considerable size; therefore, they are frequently diagnosed at an advanced stage with compressive symptoms, such as constipation, urinary disturbance, or lower limb edema.^2,4)^ Tumor size in SFTs varies widely depending on location, with reported diameters ranging from 1 to 400 mm. However, pelvic tumors exceeding 200 mm are relatively uncommon, highlighting the exceptional size in our case.^2,5)^

Because pelvic SFTs are typically hypervascular, controlling intraoperative bleeding is one of the greatest surgical challenges. Preoperative TAE has been reported to reduce intraoperative blood loss in some cases, particularly when a dominant feeding artery is identified.^5,6)^ However, in the present case, the tumor was supplied by multiple arteries, including the bilateral internal iliac, right external iliac, lumbar, and inferior mesenteric arteries, rendering selective embolization technically difficult. Therefore, we selected intraoperative balloon occlusion of the bilateral internal iliac arteries as an alternative method for vascular control. Occlusion at the abdominal aortic or common iliac level was avoided because prolonged or repeated occlusion carries limited ischemic tolerance.^7)^ Partial or staged embolization targeting selected feeding arteries was also considered. However, because no dominant feeder was identified and extensive collateral circulation was present, such an approach was unlikely to achieve a meaningful reduction in intraoperative bleeding. Balloon occlusion has also been used as an adjunctive strategy for resection of other giant hypervascular pelvic tumors, including SFTs and sacral or pelvic neoplasms, particularly when embolization is not feasible, to temporarily reduce arterial inflow and facilitate surgical progression.^7,8)^ Notably, even in these reports, substantial intraoperative blood loss has been described, indicating that balloon occlusion does not provide complete hemostatic control but rather serves to facilitate tumor resection under otherwise prohibitive conditions. This technique has been reported to facilitate bleeding control and allow safer surgery in cases of large cervical myomas where the operative space is limited; however, its application in SFTs has rarely been reported, only in limited cases.^8,9)^ In our patient, this approach provided effective temporary modulation of the pelvic blood flow and contributed to the feasibility of tumor resection.

Despite these measures, persistent venous oozing from draining veins connected to the internal iliac vein necessitated staged OAM using an Abthera device. Although OAM is generally used in patients with severe abdominal trauma, abdominal compartment syndrome, or uncontrollable peritonitis,^10,11)^ it was adopted in the present case not as routine practice but as a safety-oriented, staged strategy. Given the prolonged operative time, massive transfusion, and concern that immediate definitive closure under these conditions might exacerbate bleeding or compromise physiological stabilization, temporary packing and OAM were considered the safest option. Reports on its use in oncological surgery are scarce. In our case, initial hemostasis was achieved with compression and OAM, allowing physiological stabilization. A planned second-look operation performed on the following day confirmed the absence of active bleeding, and additional hemostasis using a topical hemostatic agent (Surgiflo) enabled safe and definitive abdominal closure. The combination of intraoperative balloon occlusion and staged OAM, as demonstrated here, has rarely been reported in the context of pelvic SFT and highlights a practical strategy for managing persistent venous oozing in complex hypervascular pelvic tumor surgery.

Histologically, the tumor exhibited spindle cell proliferation and low mitotic activity. Immunohistochemically, nuclear positivity for STAT6 confirmed the diagnosis of SFT, consistent with the *NAB2–STAT6* fusion gene.^12)^ Interestingly, CD34, which is reported to be positive in approximately 85%–95% of SFTs,^13)^ was negative in our case. Although CD34-negative but STAT6-positive SFTs have been reported, CD34-negative SFTs exhibit malignant features or dedifferentiation more frequently than their CD34-positive counterparts.^14,15)^ These findings underscore the diagnostic importance of STAT6 immunostaining, particularly in atypical cases with unusual immunoprofiles.^12,13)^

Although the patient remained recurrence-free at 1-month, long-term follow-up is essential. Previous studies have shown that even histologically benign-appearing SFTs can recur or metastasize many years after resection, with late recurrences reported up to 10–20 years postoperatively.^16,17)^ Mitotic index, necrosis, and Ki-67 labeling index have been identified as important prognostic factors for recurrence.^16)^ In the present case, the tumor exhibited necrosis and a moderate Ki-67 index of 6%, indicating the need for careful long-term observation. Moreover, PET-CT has been reported to be useful for assessing CD34-negative SFTs.^18)^ In the present case, postoperative PET revealed no abnormal uptake, supporting the likelihood of an R0 resection. Nevertheless, extended imaging surveillance remains mandatory, given the potential for late recurrence.

Our experience highlights several important clinical implications. Giant pelvic SFTs are exceedingly rare and pose formidable surgical challenges due to their hypervascularity and anatomical complexity. Intraoperative balloon occlusion provides a useful means of temporary vascular control when preoperative TAE is technically infeasible owing to multiple feeding arteries. Furthermore, staged OAM offers a valuable adjunct when immediate definitive closure is unsafe owing to persistent bleeding. Taken together, these strategies underscore the importance of individualized surgical planning in achieving safe resection of hypervascular pelvic tumors. Together, these techniques were applied as a staged strategy allowing temporal separation of arterial inflow modulation and venous bleeding control in complex hypervascular pelvic surgery.^8,10,11)^ However, this case does not represent an ideal or low-risk surgical course. Even under high-risk conditions with substantial bleeding, a staged and individualized strategy may allow complete tumor resection with acceptable perioperative outcomes.

## CONCLUSIONS

We report a rare case of a giant pelvic floor SFT with CD34 negativity and STAT6 positivity. Complete tumor resection was ultimately achieved through intraoperative balloon occlusion of the internal iliac arteries, combined with staged OAM. This case highlights the usefulness of individualized surgical strategies, including temporary vascular control and planned second-look procedures, for the management of complex pelvic tumors.

## SUPPLEMENTARY MATERIALS

Supplementary Figure 1 Additional preoperative findings. (A) CT showing bilateral hydronephrosis caused by ureteral compression (red arrows). (B–D) Angiographic images demonstrating additional feeding arteries arising from the right external iliac (B), lumbar (C), and inferior mesenteric arteries (D). Red arrows indicate the tumor in panels B–D.

Supplementary Figure 2 Immunohistochemical findings (×100) showing various tumor markers.
