# Supplementary figures and images for: Complete Resection of a Giant Hypervascular Pelvic Floor Solitary Fibrous Tumor Using Intraoperative Balloon Occlusion and Staged Open Abdomen Management: A Case Report
Source: Surg Case Rep. 2026 Mar 5;12(1):25-0717. doi: 10.70352/scrj.cr.25-0717 (PMC12972382; doi:10.70352/scrj.cr.25-0717)

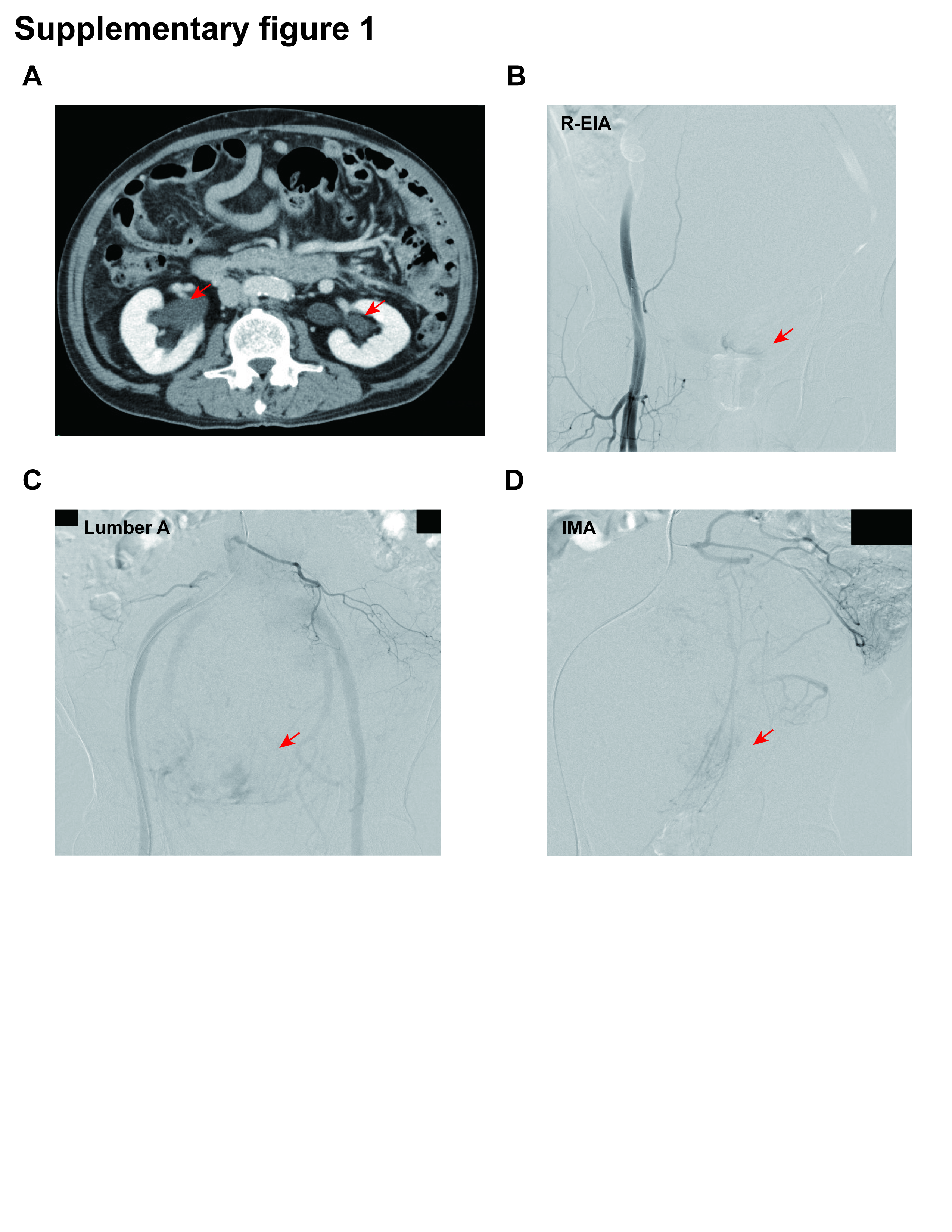

Supplement: Supplementary Figure 1 — Additional preoperative findings. (A) CT showing bilateral hydronephrosis caused by ureteral compression (red arrows). (B–D) Angiographic images demonstrating additional feeding arteries arising from the right external iliac (B), lumbar (C), and inferior mesenteric arteries (D). Red arrows indicate the tumor in panels B–D. [file scr-12-01-25-0717-s001.tif]

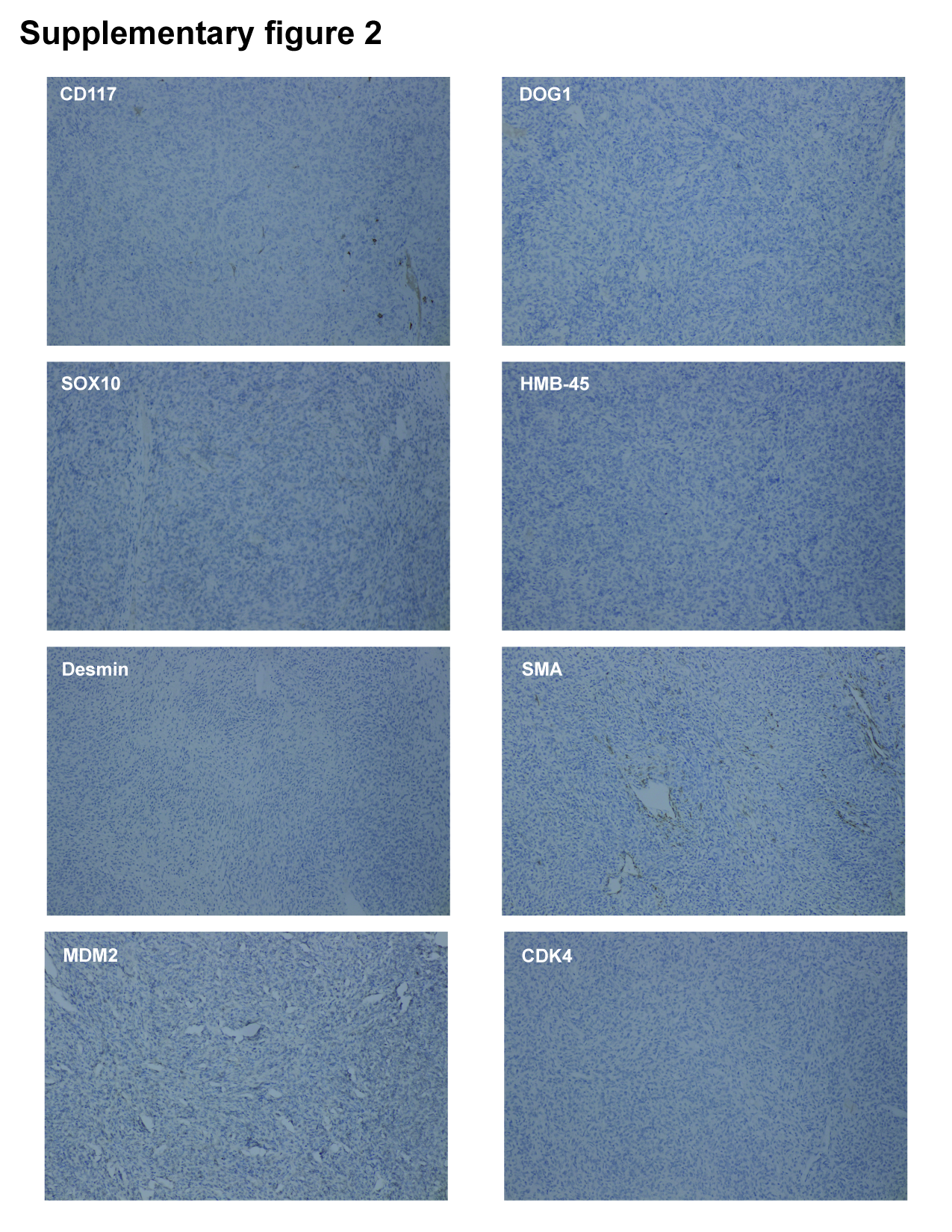

Supplement: Supplementary Figure 2 — Immunohistochemical findings (×100) showing various tumor markers. [file scr-12-01-25-0717-s002.tif]
